# Linking women experiencing incarceration to community-based HIV pre-exposure prophylaxis care: protocol of a pilot trial

**DOI:** 10.1186/s13722-019-0137-5

**Published:** 2019-03-04

**Authors:** Susan E. Ramsey, Evan G. Ames, Lauren Brinkley-Rubinstein, Anne M. Teitelman, Jennifer Clarke, Clair Kaplan

**Affiliations:** 1 Department of Psychiatry and Human Behavior, The Warren Alpert Medical School of Brown University, Providence, RI USA; 2 Department of Medicine, The Warren Alpert Medical School of Brown University, Providence, RI USA; 3 Rhode Island Hospital, Providence, RI USA; 4 Department of Social Medicine, University of North Carolina, Chapel Hill, NC USA; 5 Center for Health Equity Research, University of North Carolina, Chapel Hill, NC USA; 6 University of Pennsylvania School of Nursing, Philadelphia, PA USA; 7 Rhode Island Department of Corrections, Cranston, RI USA; 8 Department of Obstetrics and Gynecology, The Warren Alpert Medical School of Brown University, Providence, RI USA; 9 Planned Parenthood of Southern New England, Providence, RI USA

**Keywords:** Pre-exposure prophylaxis, HIV prevention, Linkage to care, Women experiencing incarceration

## Abstract

**Background:**

Women experiencing incarceration (WEI) engage in high rates of sex- and drug-related behavior that places them at risk for HIV. Pre-exposure prophylaxis (PrEP) is an efficacious means of reducing HIV acquisition. There is a general lack of knowledge regarding PrEP among women at elevated risk, and only a small percentage of at-risk women are currently engaged in PrEP care. The period of incarceration represents an opportunity to identify at-risk women, initiate PrEP during incarceration, and establish linkage to community-based PrEP care upon release from incarceration. Further, post-release is a time period that is particularly risky, and there are numerous barriers, including substance use, that may impede linkage to community-based care in the absence of intervention. The current protocol describes plans for the development and pilot randomized controlled trial (RCT) of an intervention to promote PrEP uptake during incarceration and facilitate linkage to community-based PrEP care post-release.

**Methods/design:**

The motivational interviewing-navigation (MI-NAV) study intervention is being developed, refined, and tested over three phases within the framework of the social ecological model. All phases of the study are being conducted at a women’s correctional facility and community-based PrEP provider located in the Northeastern region of the United States. Phase 1 consists of individual qualitative interviews to be conducted with key stakeholders (n = 6–10) from the community-based PrEP care site and (n = 6–10) from the women’s correctional facility, as well as with (n = 18–30) WEI. Recruitment for Phase 1 was initiated in November 2017. In Phase 2, MI-NAV will be piloted with a small cohort (n = 8–12) of WEI and will be refined based upon participant feedback. During Phase 3, a pilot RCT of MI-NAV and a standard of care condition will be conducted with 80 WEI. RCT participants will complete baseline and follow-up assessments 1, 3, and 6 months post-release. The primary study outcome is linkage to community-based PrEP care, verified via medical records.

**Discussion:**

This study will develop and evaluate a psychosocial intervention (MI-NAV) to promote PrEP uptake and facilitate linkage to community-based PrEP care among women at-risk for HIV. It is expected that, as a result of this project, the feasibility, acceptability, and preliminary efficacy of MI-NAV will be determined. If found to be efficacious, this intervention has the potential to reduce HIV acquisition in a high-need, underserved community.

*Clinical trial registration* NCT03281343

## Background

The criminal-justice involved population in the United States (US) is among the most vulnerable and heavily impacted by HIV; women experiencing incarceration (WEI) have been found to be as much as 15 times more likely to be HIV infected than women in the general population [1]. They have also consistently been found to report high levels of HIV risk behavior. A large study of female jail detainees found a 24.3% rate of weekly sex exchange in the past year and an 18.8% rate of history of injection drug use [2]. Similarly high rates of HIV-related sex and drug risk behavior have been found in other studies of WEI (e.g., [3]).

The period immediately following release from incarceration may be a particularly high risk period for HIV-related risk behavior. Binswanger et al. [4] observed consistently high rates of unprotected sex 2 weeks and 3 months post-release among a sample of women. Compared to men, women recently released from prison reported engaging in a significantly greater average number of HIV sex risk behaviors (2.31 vs. 4.73) and HIV drug risk behaviors (.07 vs 2.72), within the past month [5]. Qualitative interviews with recently incarcerated individuals revealed that both sex and drug risk behaviors were prevalent during the post-release period, the highest rates of HIV-risk behaviors occurred during the first few days post-release, there was a general lack of knowledge of HIV, and there were significant barriers to accessing health care and medications post-release [6]. Therefore, there is a strong need for effective interventions that reduce HIV risk among this population, especially during the high risk period immediately after release from incarceration.

One potential approach to addressing the HIV epidemic among at-risk women is through the use of pre-exposure prophylaxis (PrEP) [7, 8]. PrEP is a prevention intervention that currently entails the daily use of a single-tablet combination antiretroviral medication (emtricitabine/tenofovir) by HIV-uninfected individuals and is effective at preventing HIV seroconversion when taken every day [9–11]. For example, Partners PrEP demonstrated between 86 and 90% risk reduction for acquiring HIV in individuals with detectable levels of the study drug (i.e., PrEP) in their blood [11]. In addition, a 70% risk reduction in HIV infection was observed in adherent participants of the Bangkok Tenofovir Study [9].

While PrEP may be an effective strategy for preventing HIV acquisition in at-risk women, there has been little uptake of PrEP among women in the US. Indeed, PrEP prescriptions for women accounted for only 3% of all PrEP prescriptions in a national sample of persons with commercial health insurance in the US through 2014 [12]. Medicaid data from the state of New York tell a similar story, with sharp increases over the last 5 years in the number of PrEP prescriptions for men and only modest increases for women [13]. A chief contributor to the underutilization of PrEP among US women appears to be a general lack of knowledge regarding PrEP and a limited ability to accurately assess their level of risk, even among women who are at elevated risk for HIV [14–17]. However, there is significant interest in PrEP among at-risk women following psychoeducation regarding PrEP care, if barriers such as cost and accessibility can be addressed [15, 16]. In fact, in a recent study conducted by Rutledge and colleagues [8], 90% of WEI who were eligible for PrEP claimed they would try PrEP if their provider recommended a prescription.

One potential approach to improve PrEP uptake among at-risk women is Motivational Interviewing (MI) [18, 19]. MI is a collaborative, nonconfrontational approach to discussing and facilitating behavior change. HIV risk reduction interventions combining MI and skills training have been found to reduce HIV risk behavior among at-risk women [20, 21]. Further, MI has been found to significantly reduce unprotected intercourse and needle sharing among recently incarcerated women at risk for HIV [22]. A relatively recent systematic review of studies examining HIV risk reduction interventions among adults with criminal justice involvement found that MI shows promise in this population [23].

Formerly incarcerated women face individual-, interpersonal-, community-, and structural-level barriers to accessing community-based care post-release. Substance use and depression, which are highly prevalent within this population, are linked to poor treatment engagement and adherence [24–27]. Stigma, lack of transportation, and cost of care also hamper their ability to access treatment services in the community [28, 29]. Facilitators of care include being linked to community-based care, having appointments scheduled upon release, and receiving health education during incarceration [29]. Consistent with these findings, persons living with HIV who are linked to care at the time of release from incarceration are more likely to have a regular source of care compared to those who do not receive this service [30].

A potential strategy to more effectively link women recently released from incarceration to PrEP care services is through the use of patient navigators (NAVs). Traditionally, NAVs are individuals who help patients navigate the complex landscape of the healthcare system. This often takes the form of communicating a patient’s concerns with their healthcare provider, assisting patients with scheduling appointments, arranging transportation to appointments, and connecting patients with other necessary resources (e.g., housing, clothing, translation services). NAVs have been used since 1990 as a means of increasing health screenings and linkage to services. This model has been tested most commonly in oncology and is a proven evidence-based practice for linking and engaging at-risk populations to treatment [31]. Bradford et al. [32] reported the NAV model as having “promise for improving access to HIV care” and reducing health disparities among HIV-infected disadvantaged populations. However, little scientific advancement has occurred over the past decade in examining the effectiveness of culturally-tailored NAV interventions among HIV-infected populations. Further, no research, to date, has examined the utility of NAVs for linkage to PrEP among at-risk populations.

However, NAV interventions have been used to link women released from incarceration to other types of community-based care. For example, Scott and Dennis [33] evaluated the efficacy of monthly sessions with a “Linkage Manager” for women with substance problems during the 90-day period post-release from incarceration. Linkage Managers used MI with participants to provide feedback about their behavior, discuss barriers to making a change, and discuss motivation to change behavior. They also scheduled treatment appointments for participants and accompanied them to intake appointments. Participants who were assigned to receive the Linkage Manager sessions were more likely to participate in substance use disorder (SUD) treatment, return to treatment sooner, and be abstinent from alcohol and drugs, relative to participants in a “reentry as usual” control condition.

The conceptual framework that undergirds the present study is the social ecological model (SEM) [34]. The SEM has a focus extending beyond the individual, taking a crucial stance that shifts responsibility for reducing health inequalities away from individuals onto social and structural factors and the systems in which individuals are situated. Recently, the “Ecological Model of Factors that Impact Pre-Exposure Prophylaxis Attitudes and Uptake among Black MSM” was developed in response to a lack of multi-level PrEP research [35]. This model makes explicit the need to understand how structural, social, and individual factors all combine to affect PrEP uptake. Building on this model in the current study, the SEM will be used as the lens to understand how incarceration affects an at-risk woman’s behavior, relationships, risk environment, and subsequent ability to initiate and adhere to PrEP care post-release.

### Present study

**Fig. 1:**
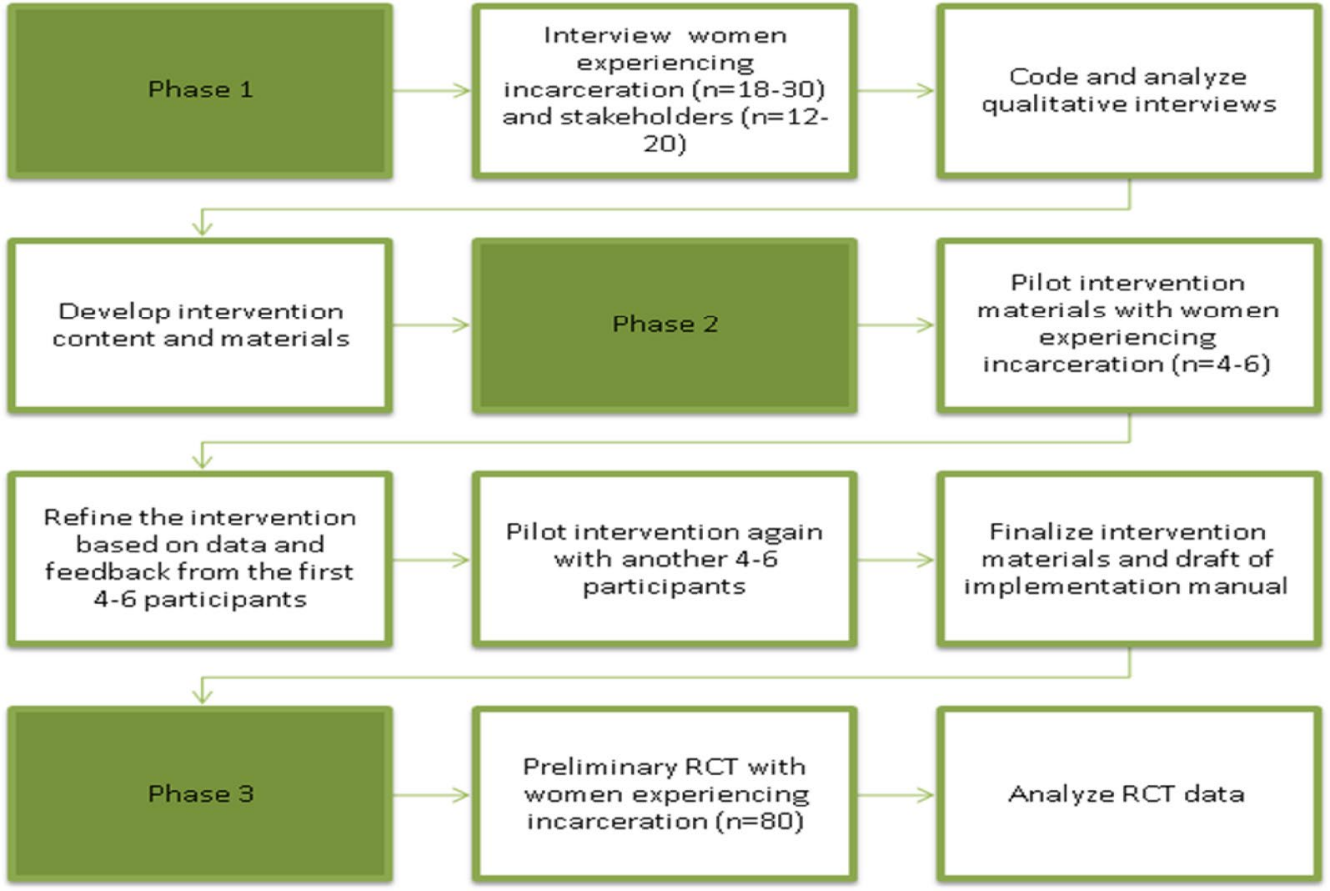
Brief description of the components and flow of the study phases

This project will address the great need to engage women at risk for HIV in PrEP care, capitalizing on a period of incarceration as an opportunity to reach a high-need, underserved population. While incarceration is a difficult time in women’s lives, it can also be a time of opportunity to obtain healthcare that they might not otherwise obtain and to focus on rebuilding a post-incarceration life with some ability to concentrate on themselves in the absence of other competing demands. We will combine an MI intervention to promote PrEP initiation during incarceration, followed by a NAV intervention to facilitate linkage to community-based PrEP care upon release from incarceration among at-risk, cisgender women, referred to as the motivational interviewing-navigation (MI-NAV) intervention. This study has 3 specific aims and will be conducted over 3 phases. The first aim is to develop the intervention materials, the second is to test the intervention and make modifications as necessary, and the third is to complete a pilot RCT comparing MI-NAV to a control condition. The aims of the study coincide with the study phases. The study was designed in this manner so that there are multiple points of feedback and refinement, prior to launch of the pilot RCT (see Fig. 1) [36, 37]. Phase 1 will consist of individual interviews with WEI at risk for HIV and with key stakeholders (e.g. administrators, health care providers, social/case workers) at a correctional facility and a community-based PrEP care site. The information gathered from these interviews will inform the development of intervention materials and an implementation manual. In Phase 2, the intervention materials and implementation approach will be tested with WEI who are at risk for HIV. Feedback from pilot participants, study staff, and stakeholders will guide refinement of the intervention and implementation approach. In Phase 3, we will conduct a pilot RCT of at-risk WEI comparing MI-NAV to a control condition that approximates standard-of-care (SOC). Data will be collected on successful linkage to PrEP care, which is operationalized as receiving a prescription for PrEP within 3 months post-release from incarceration. Data will also be collected on the acceptability and feasibility of the intervention and implementation approach.

## Methods/design

### Participants and setting

A women’s correctional facility and a community-based PrEP care site, both located in the Northeastern US, will serve as the recruitment and implementation sites for this study. The correctional facility is an integrated, statewide jail and prison that houses all females awaiting trial and/or serving a sentence. On average, there are 179 new female incarcerations per month at this facility. In 2015, 2150 women were incarcerated, and over 80% were sentenced to < 6 months. All inmates (sentenced and awaiting trial) are screened and referred, as deemed appropriate, to SUD treatment, which uses a four-tier model of treatment intensity (from most to least intensive: modified residential therapeutic community, day treatment, counseling groups, and peer support). In addition, the facility offers in-house medications for opioid use disorder (OUD) as necessary. The medication provided is determined clinically, based primarily on past experiences, patient preference, and logistical considerations. This correctional facility is working to establish a Gilead Medication Assistance program to offer PrEP to inmates. The community-based PrEP care site is a non-profit health center that offers a wide range of health care services including screening and testing for STIs, HIV testing and counseling, family planning services, reproductive healthcare for women and men, and PrEP care. This health center is currently staffed by 4 physicians, 2 full-time and 7 part-time nurse practitioners and physicians’ assistants, and 3 nurses.

Six to ten key stakeholders will be recruited from each of the sites to participate in the individual interviews. Study staff will attend staff meetings at the sites to introduce the study to stakeholders and invite their participation in the individual interviews. Participation will not be required by their respective employers, and there will be no occupational consequences for completing or not completing an individual interview. No data collected from stakeholders will be shared with their employers. Once a potentially eligible staff member has been identified, a research assistant will contact them, describe the nature of the interview, and coordinate a time to conduct the individual interview if the individual is eligible and interested in participating in the study. To participate, stakeholders must have been employed by either of the sites as an administrator, health care provider, or social worker/case manager for at least 6 months, be at least 18 years of age, and be able to understand and speak English and to provide written and verbal informed consent.

At-risk women will be recruited from the correctional facility for all phases of the study (Phase 1: n = 16–30; Phase 2: n = 8–12; Phase 3: n = 80). Announcements about the study will be made during group meetings by a trained research assistant, flyers about the study will be posted at the facility, and advertisements for the study will be included in facility newsletters. It will be made clear that participation is completely voluntary and that there are no consequences to legal status for participating or not participating. If an individual is interested in participating, the research assistant will set up a time to discuss the study with them further and screen them for eligibility. Study eligibility will be determined via a screening interview, medical record review, and consultation with correctional facility staff. Eligibility criteria include: (1) female at birth; (2) at least 18 years of age; (3) not currently pregnant; (4) risk behavior prior to incarceration that meets CDC indications for PrEP [38]; (5) it is clinically appropriate, within CDC guidelines, to initiate PrEP [38]; (6) likely to be incarcerated for < 6 months; (7) able to understand and speak English and to provide written and verbal informed consent. For this population, CDC indications for PrEP include: HIV-positive sexual partner, recent bacterial STI (gonorrhea or syphilis), high number of sex partners, history of inconsistent or no condom use, commercial sex work, residence in high HIV prevalence area or network, HIV-positive injecting partner, or sharing injection equipment [38].

### Procedures

#### Phase 1: individual interviews

Individual interviews will be conducted with the target population and key stakeholders to inform the intervention content and materials as well as the implementation approach. All participants will be asked to volunteer their time for this phase of the study. The individual interviews will be conducted in a private space and will last 60–90 min. After each interview, the research team will debrief and discuss emerging themes. We will regularly review saturation of key topics and conduct additional individual interviews if more information is warranted.

**Fig. 2:**
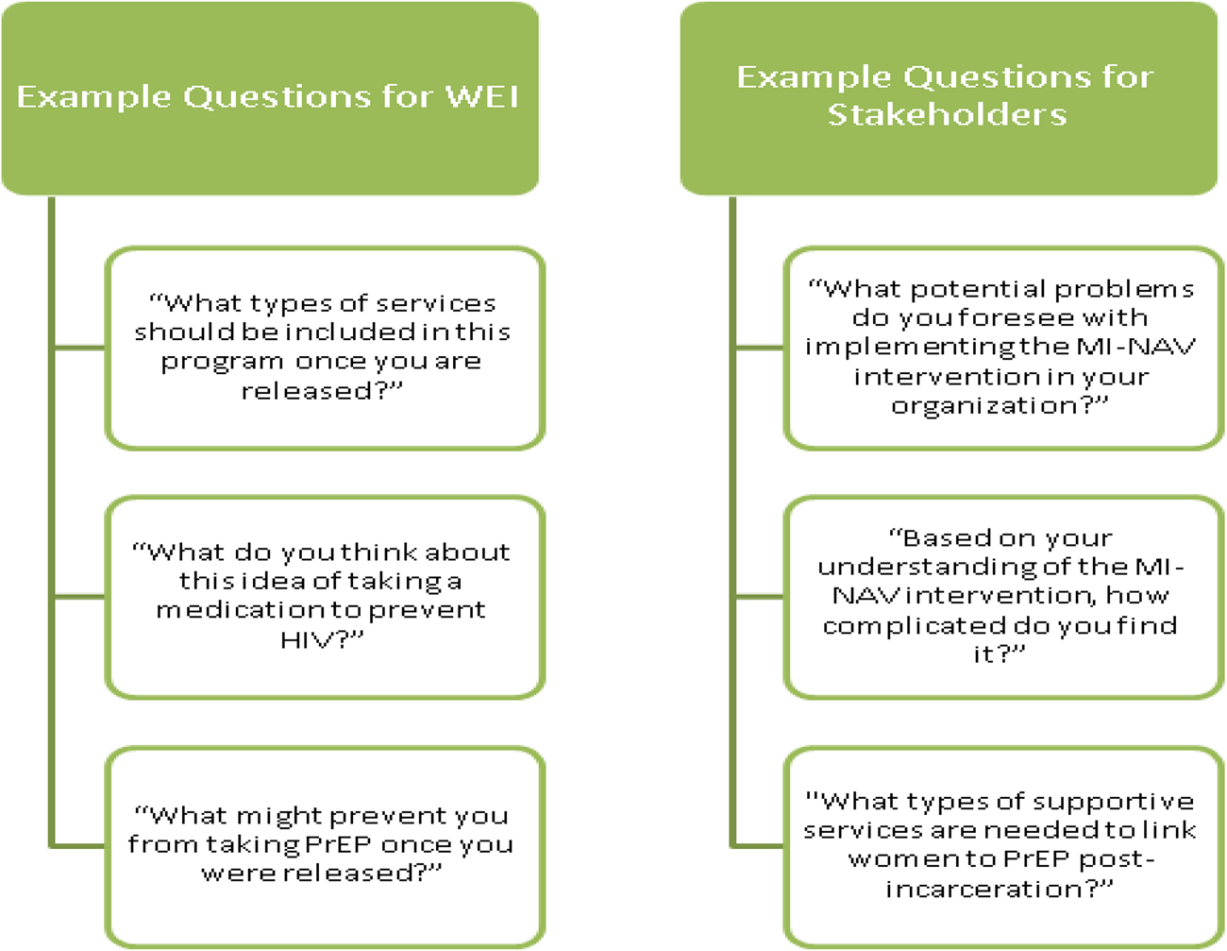
Examples of questions from individual interviews in Phase 1

During the target population interviews, we will collect quantitative data regarding demographic information and HIV-related sex and drug risk behavior during the 6 months prior to incarceration, based on CDC guidelines for PrEP indications for women. This information will be used to stratify the sample by type of risk behavior (n = 6–10 women with sex risk only, n = 6–10 women with drug risk only, and n = 6–10 women with sex and drug risk) and to perform analyses that examine differences in qualitative data. Interviews with the target population will include the following topics: (1) effect of incarceration on behavior, relationships, and the risk environment; (2) knowledge, interest, and attitudes regarding PrEP; (3) perceived need for PrEP based on certain HIV risk behaviors; (4) perceived barriers and concerns to initiating PrEP during incarceration and linking to PrEP care post-release; (5) perceived facilitators to PrEP care and suggestions for overcoming barriers to PrEP care at the correctional facility and post-release; (6) strengths and limitations of the proposed MI-NAV content and structure and suggestions to improve acceptability, feasibility, and efficacy of MI-NAV (see Fig. 2 for example questions).

During the stakeholder interviews, quantitative data regarding demographic information, length of employment, and position will be collected. Individual interviews with key stakeholders will cover: (1) implementation barriers and facilitators at the correctional facility and post-release; (2) strengths and limitations of the proposed MI-NAV content and structure and suggestions to improve acceptability, feasibility and efficacy of MI-NAV; (3) knowledge of PrEP and perceptions of its utility (see Fig. 2 for example questions).

##### Coding and analysis

All individual interview sessions will be audio-recorded and transcribed. The qualitative data from the target population and key stakeholders will be triangulated [39] and analyzed using thematic analysis [40]. This strategy will allow the research team to develop themes from the research questions and the narratives provided by the participants [41]. Research questions include: (1) What are the perceived barriers and facilitators to initiating PrEP during incarceration and linking to community-based PrEP care upon re-entry into the community among cisgender women? (2) How do certain risk behaviors, such as injection drug use, impact the perceived need for PrEP? (3) What are the perceived barriers and facilitators to PrEP adherence and retention in care during incarceration and in community-based care among women? (4) What should be the content and structure of an intervention during incarceration, at the time of release, and post-release to maximize PrEP care among at-risk women? The research team will develop a preliminary coding structure based on the interview scripts. After each debrief session following the completion of an interview, the coding structure will be updated as repeating themes emerge from the data. This will lead to the creation of a final coding structure, in which thematic categories will be refined, merged, or subdivided into subcodes. Then, two independent coders will use the final coding structure to double code the transcribed interviews and an inter-coder reliability estimate will be computed. Standard analysis techniques will be utilized, including open coding, axial coding, marginal remarks, and memo-writing [40]. The data acquired from these methods will be used to inform intervention materials and the implementation approach.

#### Phase 2: pilot test of intervention materials and draft of implementation manual

All intervention materials will be created after the conclusion of the individual interviews in Phase 1. The SEM and feedback we receive from WEI and stakeholders in Phase 1 will create a framework that informs the development of intervention materials. Once developed, we will conduct a preliminary test of the intervention with a total of 8–12 WEI who are at risk for HIV and meet all other study inclusion criteria. During this phase, participants will complete a baseline interview while incarcerated, receive the MI-NAV intervention, and participate in an individual interview post-release. Participants will not be compensated for the baseline interview while incarcerated but will receive a $50 gift card for completing the individual interview post-release. At the conclusion of this phase, a final version of clinician and patient manuals, as well as training materials, will have been developed for the MI-NAV intervention.

##### MI-NAV intervention

While the specific content, structure, and implementation approach for MI-NAV will be guided by the SEM and data from the individual interviews conducted during Phase 1 and Phase 2, a basic outline of the intervention has been developed. Our initial plan is to deliver MI-NAV in two segments. The first segment will be aimed at promoting uptake of PrEP during incarceration. The second segment will be aimed at linking at-risk women to community-based PrEP care upon release from incarceration. In the intervention’s current form, our plans are for the first segment to consist of an in-person 50-minute session with a study interventionist. Master’s-level clinicians will be hired as study interventionists and will be trained to deliver intervention materials by the study’s principal investigator. During this session, the interventionist will review how certain behaviors, such as injection drug use, increase HIV risk and will employ motivational interviewing techniques to explore the participant’s interest in starting PrEP while incarcerated. If the participant expresses an interest in beginning PrEP care, the interventionist will set up an appointment for the participant to initiate PrEP care with a provider from the correctional facility. The second segment of the intervention will begin 2 weeks before the participant is released from incarceration. It will first consist of a second in-person, 50-minute session with the same study interventionist. During this session, the interventionist will employ motivational interviewing techniques to either explore the participant’s interest in continuing PrEP care in the community once they are released or again discuss initiating PrEP care if the participant has not done so at this point. Strategies for overcoming obstacles to connecting to care in the community will also be discussed. When appropriate, referrals to community-based SUD treatment will be provided. If the participant wishes to continue or start PrEP care once they are released, the interventionist will facilitate the initial PrEP appointment scheduling with the community-based PrEP care provider and accompany the participant to the initial appointment. Following release, the same interventionist will conduct either a monthly face-to-face or telephone check-ins with the participant, whichever is preferred by the participant, for 6 months. These check-ins will be brief, but will be allowed to last up to 50 min if needed. They will each consist of: (1) a review of recent HIV risk behavior, including substance use, and referrals to community-based SUD treatment when appropriate; (2) discussion of PrEP and problem solving for barriers to PrEP adherence; (3) discussion of other HIV risk reduction strategies.

##### Refinement of MI-NAV

**Fig. 3:**
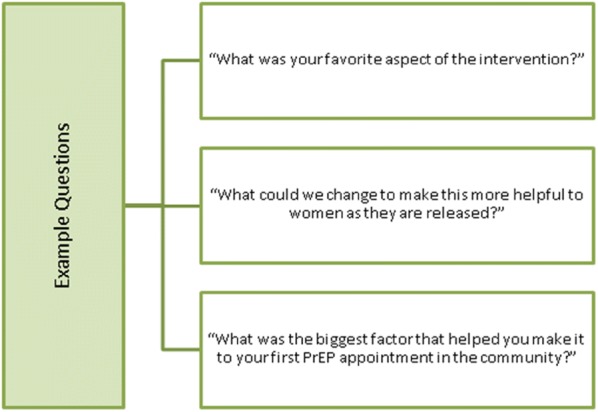
Example questions from individual interviews post-release in Phase 2

The purpose of the individual interviews post-release will be to elicit feedback about the MI-NAV intervention, including any strengths and/or limitations perceived by the pilot participants (see Fig. 3 for example questions). The same coding and analysis methods described above will be utilized for the qualitative data collected during this phase of the study. Specifically, we will look for repeating themes from the narratives provided by the first 4–6 pilot participants and make adjustments to the intervention accordingly. This entire process will then be repeated again with another 4–6 pilot participants, leading to a final version of the MI-NAV intervention.

#### Phase 3: pilot RCT

For the pilot RCT, 80 participants will be recruited and assigned to a condition using a 3:1 ratio, with 60 participants assigned to MI-NAV and 20 participants assigned to SOC. Participants in the SOC condition will also meet with a study interventionist twice while incarcerated. During the first session, SOC participants will receive a pamphlet regarding PrEP during incarceration and will be informed that they can initiate PrEP by speaking with a provider from the correctional facility. Mirroring MI-NAV, the second session will occur 2 weeks before the participant is released from incarceration. This session will involve a referral to community-based PrEP care if the participant is interested and referral to community-based SUD treatment when appropriate. Participants will be randomly assigned to condition using urn randomization; the urn blocking variable will be baseline level of HIV risk behavior as assessed by the HIV Risk Assessment Battery (RAB) [42]. The 3:1 randomization ratio will allow us to maximize the information gained about the MI-NAV intervention while including a comparison condition. RCT participants will complete a baseline interview while incarcerated and follow-up interviews at 1, 3, and 6 months post-release from incarceration. Baseline and follow-up interviews will be conducted by research assistants who will be blind to participants’ assigned condition. RCT participants will receive gift cards in the amount of $25, $30, and $50, respectively, for the 1-, 3-, and 6-month follow-ups (see Table 1 for timing of Phase 3 protocol elements). No compensation will be provided for the baseline interview or sessions/check-ins with the interventionist.

**Table 1:** Timing of participant enrollment, receipt of intervention, and assessment activities during Phase 3

Timepoint	Enrollment	Baseline	Post-allocation		
	− t1	0	+ 1 month	+ 3 months	+ 6 months
Enrollment					
Eligibility screen	X				
Informed consent	X				
Allocation		X			
Interventions					
MI-NAV					
SOC		X			
Assessments					
Primary outcomes			X	X	X
Secondary outcomes			X	X	X
Other outcomes		X	X	X	X

##### Measures

The primary outcome for this study is linkage to community-based PrEP care post-release. Secondary outcomes include PrEP initiation during incarceration, PrEP adherence, and retention in PrEP care. HIV and other STI test results will be extracted from medical records, and an HIV test will be performed for research purposes at the 6-month follow-up. Acceptability and feasibility of the intervention will also be assessed. Additional quantitative data will be collected during the baseline and follow-up assessments (see Table 2).

**Table 2:** Schedule of assessments

Quantitative measures	Screen	Baseline	1-, 3-, and 6-months follow-up
Inclusion/exclusion criteria (including negative HIV and pregnancy test)	X		
Descriptive information			
Demographics		X	X
PrEP care initiation during incarceration and linkage to care in community			X^a^
PrEP care retention			X^a,b^
PrEP adherence			
Dried blood spot test			X^b^
Self-reported adherence			X
HIV test results		X^a^	X^c^
Other STIs, hepatitis B, hepatitis C test results		X^a^	
Potential moderators of treatment effects			
Timeline followback for alcohol and drug use (TLFB)		X	X
Timeline followback for HIV sex and drug risk behavior and HIV risk assessment battery (RAB)		X	X
Center for Epidemiologic Studies Depression Scale (CES-D)		X	X
Treatment received: treatment services review		X	X
Use of medications/pregnancies		X	X
Intervention acceptability			X^c^
Intervention feasibility			X^c^
Reasons for linkage/non-linkage to PrEP Care/risk reduction			X^c^

^a^Extracted from participants’ medical records

^b^Collected only at 3 and 6 months

^c^Collected only at 6 months

##### PrEP care initiation and linkage to care

Information will be extracted from participants’ medical records at the correctional facility regarding whether PrEP was prescribed and whether PrEP was administered each day during the period of incarceration. Linkage to community-based PrEP care is operationalized as receipt of a prescription for PrEP from a community-based provider within 3 months of release from incarceration, verified through medical record data extraction. This time window was selected because it is consistent with the CDC’s guidelines for PrEP follow-up visits (every 3 months) [38].

##### PrEP adherence and care retention

PrEP adherence will be determined by drug concentrations of emtricitabine/tenofovir in dried blood spot samples collected from participants who have been prescribed PrEP at 3- and 6-month follow-ups. The lower limit of quantification for this test, or ability to detect drug concentrations, is 10 ng/ml [43]. Values under 10 ng/ml will be treated as “undetectable.” Drug concentrations will be entered as continuous variables for data analysis. Self-reported adherence will also be assessed using a well-validated 3-item measure, known simply as the Three-Item Self-Report Measure for Medication Adherence [44]. The three items focus on the past 30 days and include: 1) an assessment of how many doses of medication were missed, 2) a self-rating of how well the participant managed to take their medication in the way they were instructed, and 3) a self-rating of how often the participant managed to take their medication in the way they were instructed. Retention in PrEP care is being defined as attendance to 3-month (± 1 month) clinical appointments in accordance with current CDC guidelines for PrEP care [38]. These guidelines include follow-up visits at least every 3 months to determine if it is clinically appropriate to continue PrEP care. These visits typically involve testing for HIV, bacterial STIs, pregnancy, and assessment of renal function. Attendance to follow-up PrEP appointments will be extracted from participants’ medical records.

##### Potential moderators of treatment effects

It is possible that certain variables may be potential moderators of treatment effects. Alcohol and drug use [24–26], HIV risk behaviors [45, 46], housing status [47, 48], and depressive symptoms [27] have been found to be associated with poor treatment engagement and medication adherence in previous research. Therefore, we will be collecting information about these variables for exploratory analyses. The Timeline Follow Back (TLFB), a calendar-assisted structured interview, will be used to collect data on the number of standard drinks consumed per day and types of drug classes used each day within a given time period [49–51]. The TLFB will also be used to assess daily HIV drug and sex risk behaviors. In addition, the HIV Risk Assessment Battery (RAB) will serve as a measure of overall HIV sex and drug risk behavior [42]. The RAB assesses the frequency of behaviors such as injection drug use, sex without a condom, sex while under the influence of substances, and sex in exchange for money or drugs. Frequencies fall on scales with response options between a range of 0 (least frequent option, depending on behavior) to 3 (most frequent option, depending on behavior). An overall risk score, which is correlated with seroconversion, is computed by adding the values corresponding with the response option (e.g. 0–3) and then dividing this total score by the highest possible score. Information on housing status will be collected, along with other demographic information, at baseline and each of the follow-up appointments. Finally, the Center for Epidemiologic Studies Depression Scale (CES-D) will be used to measure level of depressive symptoms [52].

##### Intervention feasibility and acceptability

At the conclusion of the study, we will compile a patient eligibility rate, enrollment refusal rate, rate of recruitment, and follow-up completion rate in order to evaluate the feasibility of conducting a subsequent larger scale study using this protocol. We will also compile a study dropout rate and intervention session completion rate, as indices of acceptability. In addition, the 8-item Client Satisfaction Questionnaire-Revised will be used to further evaluate intervention acceptability and feasibility [53].

##### Reasons for PrEP care linkage and non-linkage/risk reduction strategies

During the 6-month follow-up assessment, all participants will complete a brief qualitative interview in which they will be asked to reflect on factors that impacted their use or non-use of PrEP both while incarcerated and after release. Participants will also be asked about any other HIV risk reduction strategies that were employed. During analysis, participants’ responses will be stratified and examined based on level of PrEP uptake per condition.

### Planned data analysis

REDCap, a secure web application, will be used as the primary data tracking, data collection, and data management platform for all assessments. Quantitative data analyses will be conducted only on participants recruited during Phase 3 (i.e., once random assignment has begun). As a first step, the equivalence of treatment conditions with regard to key baseline variables will be assessed. This will involve comparisons of treatment conditions on demographic characteristics and baseline levels of potential treatment moderators. Should conditions differ on any characteristic, these variables will be placed in models as interactions with group assignment along with its main effect and also in a distinct model with the interaction removed. The model with the lowest AIC will be retained. Other preliminary analyses will include studies of patterns of missing data, research dropout rates, distributional properties of dependent and other measures, and correlations among outcomes measures.

Data analysis will follow a sequence designed to examine the primary outcome questions: (1) Does MI-NAV lead to higher rates of linkage to community-based PrEP care? (2) Does MI-NAV lead to better PrEP adherence, based on dried blood spot tests (DBS), compared to SOC? (3) Does MI-NAV lead to better retention in care compared to SOC? There is little expectation of observed HIV seroconversion, given the modest sample size and 6-month follow-up window. However, seroconversion rates of each condition will be examined. Analyses will also be conducted to examine the relative impact of MI-NAV versus SOC on self-reported PrEP adherence. Following the intention-to-treat principle, all randomized participants will be included in the analyses. We anticipate an attrition rate less than 10%, which will provide us with a final sample size of at least 54 participants in the MI-NAV condition and 18 participants in the SOC condition. This estimate is based on previous research with a high-risk, incarcerated population, in a geographically similar setting, which retained 96% of participants at 12-month follow-up [54]. In addition, we will employ retention strategies that increase retention rates in this population [55], including: (1) offering interview incentives that are higher than normal hourly rates of pay and providing transportation to and from interview locations; (2) conducting regular phone check-ins or sending letters between appointments to ensure contact information has not changed; (3) collecting contact information of secondary contacts, such as friends or relatives, that will know how to reach the participant; and 4) working with local criminal justice agencies, parole officers, and police departments that can help maintain or reestablish contact with participants if necessary.

Given that this is a pilot study, the primary goal is to yield a stable estimate of the effect size rather than to find statistically significant differences. The effect size estimate will be useful in planning a future RCT. We are aware of the dangers of relying exclusively on small pilot studies to gauge the promise of interventions [56]. These effect size estimates have a large standard error, and we primarily will be hoping to find a pattern of results that is supportive of MI-NAV, at which point a full scale trial will be designed to test for a clinically meaningful effect size. To provide stable odds ratios for estimates of effect size for dichotomous or categorical variables, such as PrEP care linkage, a somewhat larger sample size is required. For continuous variables, group means typically begin to stabilize around 15 participants per group. We believe that the sample size of 60 participants in MI-NAV and 20 participants in SOC, even after attrition, will allow us to evaluate the potential of MI-NAV to improve PrEP linkage while maximizing the number of participants in the MI-NAV condition in order to fully assess the feasibility and acceptability of the intervention.

#### Primary RCT analyses

Tests of the effects of treatment on the primary outcome variable (linkage to community-based PrEP care) and secondary outcomes (PrEP DBS adherence and retention in care at 3 and 6 months) will be conducted using a fractional logit model [57] estimated by Generalized Estimating Equations (GEE) [58–60]. GEE is a quasi-likelihood estimation method of repeated measures analysis for appropriate modeling of covariance structures when outcomes are correlated across time. Additionally, it allows for the inclusion of both categorical and continuous independent variables. While it is most common to use logistic regression to analyze dichotomous variables, the fractional logit model can be used for any fractional outcome with a range of 0–1. An advantage of GEE over ANOVA is that GEE models nesting by adjusting the standard errors of the test statistics based on the covariances (and variances) of nested observations, rather than depending on calculating differences. These variances and covariances can be modeled based on all data available. Therefore, a subject with missing data for one time point will not contribute to the variance or covariance estimates involving that time point, but their non-missing time points will be used to estimate those variances and covariances.

The primary, between groups, independent variable in the above GEE is treatment group. Variables measured at baseline will be examined using screening runs prior to primary analyses to see which of these baseline measures are most strongly associated with the outcomes (linkage to PrEP, PrEP DBS adherence, retention in PrEP care, and self-reported adherence). Those that show significant relationships with outcome will be entered as covariates in the primary analyses unless there are concerns over multicollinearity. The linear effect of time will also be included as a covariate in these analyses, as we assume that retention and adherence rates will show a tendency to decrease over time. We will also test for non-linear (i.e., linear plus quadratic) effects of time for the repeated measures, adherence and retention. Testing the time by group interaction will indicate the extent to which treatment differences are more or less pronounced over time.

Analyses will be conducted separately on two overlapping samples. Following the intention-to-treat principle, all randomized participants will be included in the first set of analyses. This is the most conservative approach and represents our main outcome analysis. Analyses will also be conducted on subjects who completed the assigned intervention, the “as-treated” analysis. Although subject to more bias, especially if attrition rates are high, this latter approach answers more directly the question of intervention efficacy by providing an estimate of the maximal effects attained by an intervention. Similar results with both approaches increases confidence in the findings.

#### Missing data

In our experience, missing data is unavoidable. However, every effort will be made to minimize and appropriately handle missing data. Follow-up data will be gathered regardless of whether the intervention was received, and follow-up will be vigorously pursued to minimize missing data. Participants will be followed independent of whether they are engaged in PrEP care. If a participant is re-incarcerated during the follow-up period, we plan to work with the correctional facility to continue to follow them and avoid loss to follow-up. In addition, we will request permission to collect data from medical records for participants who do not complete a follow-up.

Whenever possible, we will collect and summarize the reasons participants drop out of the study. We will also evaluate missing data to determine if there are mechanisms that help to explain why data are missing and will utilize multiple imputation techniques [61]. Variables collected at baseline, such as demographic information, alcohol and drug use prior to incarceration, and HIV risk behaviors prior to incarceration, will be compared between participants retained throughout the study and participants who missed follow-up appointments. To increase confidence in our findings, sensitivity analyses will be performed with and without the missing data, using imputed data from the individuals with whom we lose contact.

## Discussion

Women with criminal-justice involvement are among the most vulnerable and heavily impacted by HIV [1], consistently reporting high rates of both sex and drug risk behavior [2, 3, 62, 63]. The link between risk behavior and incarceration is unsurprising given that many of the behaviors that place women at risk for HIV, such as injection drug use and transactional sex, also put them at risk for incarceration. Further, the time immediately following release from incarceration appears to be a particularly high risk period for HIV-related risk behavior among women [4–6]. PrEP can be a highly effective means of preventing HIV infection among at-risk women [9–11]. Currently, PrEP is underutilized among US women who are at risk for HIV [12, 14]. This protocol seeks to address the great need to engage women at risk for HIV in PrEP care, capitalizing on a period of incarceration as an opportunity to reach a high-need, vulnerable population.

While this study will advance our knowledge of barriers and facilitators to PrEP care among women at risk for HIV upon release from incarceration, there are some limitations that warrant recognition. First, since the goals of the study are to establish effect size estimates and to determine the feasibility and acceptability of the intervention, a modest sample size will be recruited. Therefore, it is unlikely that we will find statistically significant differences between treatment conditions. Second, due to the pilot nature of the current study, recruitment will occur at a single correctional facility. This impacts our ability to generalize the findings to other correctional settings and locations. If the intervention appears to be effective at promoting uptake of PrEP during incarceration and linkage to PrEP care post-incarceration, future research should employ a significantly larger sample size and multi-site recruitment to address these limitations.

Despite the aforementioned limitations, this study has the potential to significantly inform the field. Since risk of acquiring HIV is disproportionate within this population relative to others due to both elevated rates of sex and drug risk behavior, it is essential to develop tailored interventions that reduce their HIV risk as they reenter the community. Therefore, engaging at-risk women in PrEP care before and after release from incarceration has the potential to reduce their risk of acquiring HIV. However, women recently released from incarceration experience many barriers to receiving care, including substance use, stigma, and lack of transportation [28, 29, 64]. To be effective, an intervention will need to consider these barriers in order to facilitate linkage with community-based treatment. If successful, the MI-NAV intervention will increase uptake and linkage to PrEP care and thereby substantially reduce HIV seroconversion among this population. More broadly, if this treatment approach is effective, it could be applied to other types of treatment post-incarceration, such as treatment for SUD.

## Abbreviations

PrEP: pre-exposure prophylaxis
MI-NAV: motivational interviewing and patient navigator intervention
SOC: standard of care
